# Scalable Manufacturing of Amino Acid‐Based Piezoelectric Biocrystal Films

**DOI:** 10.1002/smll.74257

**Published:** 2026-06-21

**Authors:** Shuting Wang, Bijay Dhungana, Anirudh Reddy Bendaram, Jiajie Sui, Chenli Jia, Fnu Joshua, Craig Neal, Ruoxing Wang, Pengfei Chen, Fengdan Pan, Sharar Muhtasim, Joseph Andrews, Wenxiao Pan, Lei Zhai, Sudipta Seal, Lih‐Sheng Turng, Xudong Wang

**Affiliations:** ^1^ Department of Materials Science and Engineering University of Wisconsin‐Madison Madison Wisconsin USA; ^2^ Advanced Materials Processing and Analysis Center (AMPAC) Department of Materials Science and Engineering University of Central Florida Orlando Florida USA; ^3^ Department of Mechanical Engineering University of Wisconsin‐Madison Madison Wisconsin USA; ^4^ Department of Chemistry University of Central Florida Orlando Florida USA; ^5^ Department of Electrical and Computer Engineering University of Wisconsin‐Madison Madison Wisconsin USA; ^6^ Nanoscience and Technology Center University of Central Florida Orlando Florida USA; ^7^ Biionix cluster College of Medicine University of Central Florida Orlando Florida USA

**Keywords:** amino acids, continuous crystallization, glycine thin films, manufacturing, piezoelectric biocrystals

## Abstract

Glycine is a promising piezoelectric biocrystal owing to its excellent biocompatibility, strong piezoelectric response, and high crystallinity. Recent discovery of polymer‐directed crystallization of glycine in an aqueous environment offers the feasibility for continuous manufacturing of this biocrystal film at the industry‐relevant level. In this paper, we report continuous production of glycine‐PVA piezoelectric biocrystal thin films by adapting a commercial polymer manufacturing apparatus with a uniform sandwiched microstructure. By tuning the feeding rate and solvent evaporation dynamics, a stable crystallization front is maintained, enabling continuous film growth over lengths of tens of centimeters, which largely matches a numerical kinetic model. The as‐produced films exhibit sandwiched microstructure, with uniform thickness, grain size, and piezoelectric coefficient across the entire area. Fifty‐six piezoelectric nanogenerator devices were fabricated on a single large‐scale film, demonstrating the feasibility of high‐throughput device production. This work establishes a stable and industrially viable strategy for the large‐scale manufacturing of piezoelectric biocrystal films, offering significant potential for mass production of biocompatible piezoelectric devices.

## Graphical abstract

This work presents a continuous manufacturing strategy for glycine‐PVA piezoelectric films through dynamically controlled crystallization. This stable and industrially viable strategy offers significant potential for mass production of piezoelectric biocrystal films and biocompatible devices.

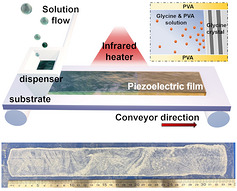

## 1 Introduction

Piezoelectric materials have been extensively investigated across inorganic single crystals [1, 2], polycrystalline ceramics [3, 4], and organic systems [5, 6]. Their ability to function as energy harvesters [7, 8, 9], sensors [10, 11], actuators [12], and therapeutic devices [13, 14] has led to rapid development of piezoelectric devices in biomedical engineering. However, the limited biocompatibility, poor degradability, and mechanical rigidity of traditional inorganic piezoelectric materials restrict their application in biological systems [15]. These challenges have prompted extensive studies of soft, biocompatible, and biodegradable piezoelectric materials that can safely work in living systems [15]. Amino acids, as natural molecular building blocks with intrinsic dipolar structures [16], represent a prospective type of candidate offering excellent biocompatibility and relatively strong piezoelectric coefficients among organic and biomaterials.

Glycine is the simplest amino acid that has been well studied because of its potential in the field of piezoelectric biomaterials [17]. The three polymorphs of glycine exhibit distinct differences in thermodynamic stability and piezoelectric performance [18]. The kinetically favored α‐glycine, which predominantly crystallizes from aqueous solution, lacks net dipole alignment and therefore exhibits no piezoelectricity. The β‐glycine possesses the strongest shear piezoelectric coefficient among three forms, but is thermodynamically metastable [19, 20, 21]. The γ‐glycine combines thermal stability with distinct piezoelectric performance, making it the most promising polymorph for functional device applications [22, 23, 24]. Currently, many studies have demonstrated the feasibility of controlling the phase of this functional biocrystal and enabling promising applications in biocompatible and degradable biomedical devices [24, 25, 26]. Further translation of the sustainable material and devices from the laboratory discoveries to clinical applications requires a scalable manufacturing strategy to produce piezoelectric biocrystals continuously in a large scale with desirable homogeneity. A recent study from our group showed a promising strategy that the γ phase can be selectively stabilized during co‐evaporation of a mixed water solution of glycine and poly(vinyl alcohol) (PVA) [23]. In this process, PVA precipitates preferentially to form thin layers at the air and substrate interfaces, where strong hydrogen bonding at the PVA‐solution interface induces the oriented crystallization of γ‐phase glycine, yielding piezoelectric films with reported d_33_ values around 5.3 pC/N. This water‐based self‐guided process opens a possibility to realize a continuous production of the piezoelectric biocrystal films, if the air‐water‐polymer interfaces can be well controlled and stabilized in a continuous fashion. In this paper, we report a scalable manufacturing strategy by adapting an industry‐level polymer fabrication process, which enables a continuous growth of glycine‐PVA piezoelectric biocrystal films. By balancing the precursor feeding and solvent evaporation on a rolling conveyor platform, this system maintains a stable crystallization front as the solution unstoppably being carried over the heating zone, allowing the crystalline film to grow continuously. The fabricated films exhibit highly uniform thickness, grain width, and piezoelectric coefficient across the entire film. This work fundamentally extends our static polymer‐guided crystallization process [23, 25] into a continuous system by coupling solution transport, substrate movement, and thermal gradients to sustain stable crystal growth kinetics under dynamic operating conditions. This work provides an industrially scalable pathway for the continuous manufacturing of amino acid‐based piezoelectric biocrystal films, providing significant technological translation potential for scaling piezoelectric biomaterials and devices.

## 2 Results and Discussion

### 2.1 Continuous Crystallization and Kinetics Study

**FIGURE 1:**
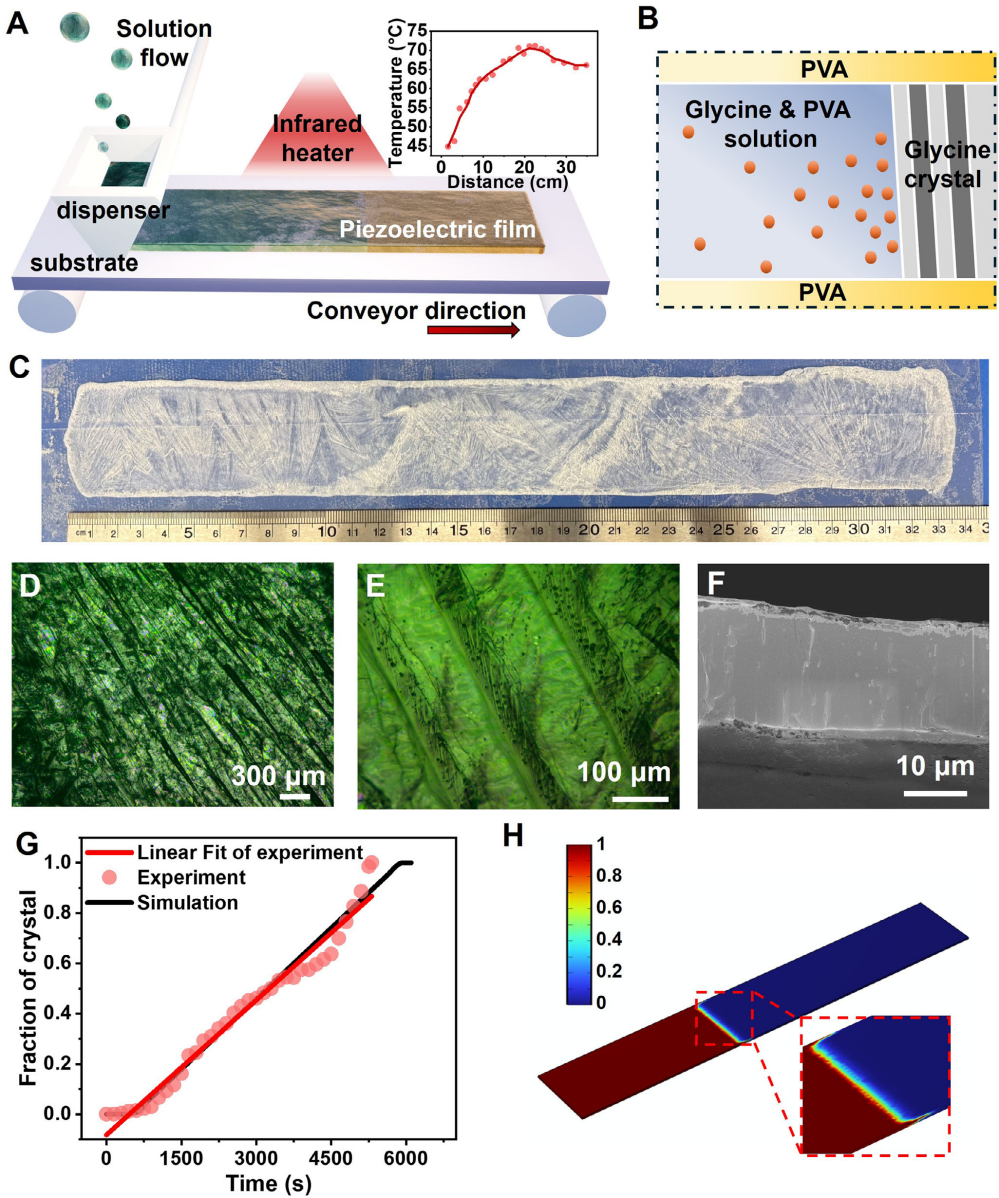
Continuous crystallization of piezoelectric biocrystal film. (A) Schematic setup and manufacturing process of glycine‐PVA films. (B) Schematic showing the crystallization of glycine crystal guided by PVA layers. (C) Photograph of a large‐scale piezoelectric glycine‐PVA film. (D) Polarized optical microscope of an as‐obtained glycine‐PVA film. (E) Zoomed‐in image from (D) showing the crystal grain alignment and surface features. (F) Cross‐sectional SEM image showing the sandwiched structure of the film. (G) Simulated (black line) and experimental (red dots) growth curve of fabrication process of glycine‐PVA film. Red solid line is the linear fitting curve of experimental data. (H) Simulated process showing the crystallization of the glycine‐PVA film from a liquid precursor film. The colormap indicates the spatial distribution of the crystalline fraction.

According to our previous study [23], a highly‐oriented γ‐glycine film could be directly precipitated from a mixture solution with 3:1 glycine‐to‐PVA ratio through controlled evaporation. Therefore, the design principle is to achieve a stable crystalline of such a mixture on a continuous scale, while the crystal‐solution interface can be constantly remained at desired crystallization conditions, while the supply of nutrient solution and the crystallization rate are dynamically balanced. The scalable manufacturing system is designed based on a flat belt conveyor system with a goal to achieve continuous crystallization of a glycine‐PVA system under identical conditions. The schematic setup is schematically shown in Figure 1A, and the actual setup is given in Figure S1. The flat belt on the conveyor was driven by an electronic motor at controlled speeds, continuously carrying the film toward collection end. A burette was applied to control the flow rate of the precursor solution. A dispenser reservoir with a narrow line‐shaped outlet was used to continuously distribute the nutrient solution. A sponge pad was attached to the dispenser outlet while making direct contact with the PET substrate; thereby, the precursor solution was uniformly distributed across the substrate with a controlled spreading width. The flexibility of PET substrate enables conformal contact with the conveyor system and is compatible with potential application on scalable roll‐to‐roll manufacturing processes, while it may introduce surface undulations under mechanical stress during processing. The solution thickness was controlled to ∼0.6 mm by adjusting both the conveyor‐belt moving speed and the solution feeding rate, which can be further tuned by applying an additional blade system (Figure S2). Furthermore, the control of precise ratio between these two rates can fabricate desired films with different thickness. An infrared heater was positioned downstream along the moving direction to establish an appropriate temperature profile for solvent evaporation and glycine‐PVA precipitation, where the maximum temperature point remained at ∼70°C (inset of Figure 1A).

As the solution film being carried toward the higher temperature zone, it was heated, and the solvent evaporated bring the solution to the supersaturation. Crystallization then occurred at the front edge of the liquid film after a relatively long nucleation process and gradually propagated toward the direction where the solution was supplied. The liquid–solid interface eventually stabilized at ∼11 cm away from feeding solution front balanced by the temperature and conveyor speed. Under this condition, along with the evaporation of water, the amphiphilic PVA precipitated first and accumulated at both air‐solution and solution‐substrate interfaces. Further evaporation of water from the top surface built a concentration gradient of glycine under the top PVA layer. Nucleation of glycine began at the supersaturated solution along the edge under the top PVA, and crystals grew with an orientation alignment [23, 25]. Figure 1B). This continuous crystallization process was shown in supplementary Movie S1, which could be sustained as the crystalline film was transported toward and collected at the collecting end. By this process, a glycine‐PVA film with a length of ∼33.5 cm was produced, as shown in Figure 1C. The film showed a visible polycrystalline domain feature, similar to those obtained from static crystallization. The edge with dense white features marked where the nucleation started, while the alternating cloudy and transparent stripes showed the partial alignment of glycine crystals. The crystalline domains exhibited a columnized morphology, with a typical width in the range of ∼40–100 µm and the length extends to several millimeters (Figure 1D). The polarized microscope also revealed a uniform color contrast across the entire crystal surface, indicating the regular orientations of the glycine crystals might be largely aligned. A zoomed‐in image further showed a nearly parallel grain boundaries with a typical grain width of ∼100 µm (Figure 1E). The dark contrast dots distributed near grain boundaries were possibly defects in PVA layers. A cross‐sectional scanning electron microscopy (SEM) image showed that the film had a typical sandwiched structure with an overall thickness of ∼20 µm (Figure 1F). The top and bottom layers were PVA polymer with the same thickness of ∼2 µm, while the middle layer (∼16 µm) was glycine with a clear crystalline feature aligning along the same orientation.

To understand the crystallization kinetics in this continuous process, we first quantified the fraction of crystalline film formed as a function of time, as shown by the red dots in Figure 1G. Under a constant temperature and feeding rate, the onset of crystallization was identified after ∼300 s from initial solution application. This incubation time was longer than a static system, which could be attributed to the slow evaporation of the solution. It took ∼450 s for the system to reach ∼1% crystallinity, which is typically defined as the starting point of crystal growth. The system then reached a fairly steady crystal growth stage in the period from 1000 to 5000 s, with a fitted linear growth rate of 1.79 × 10^−4^/s (the slope of red linear fitting curve in Figure 1G). The small deviation of the growth observed after 3500 s was possibly due to some localized re‐nucleation induced by edge or interface perturbations during the growth. Toward the end of the process, however, the growth rate gradually increased, which could be attributed to the increasing concentration of glycine in the remaining liquid. This non‐linear end region can be ignored if the process continues and thereby will not be considered in further analysis.

This continuous crystallization process was further quantified and simulated by a continuum‐scale modeling framework. This model captures multiple thermodynamic and kinetic parameters, including diffusion rate, heat transfer, evaporation rate, supersaturation level, and nucleation and secondary nucleation concentration thresholds. In this model, the crystallization is assumed to occur when the solution concentration reaches a critical threshold: the nucleation concentration of glycine is 333 mg/mL, and the secondary nucleation concentration of glycine following primary nuclei formation is 254 mg/mL [27]. A snapshot of the simulated crystallization process is shown in Figure 1H, which illustrates that crystallization initiates preferentially at both lateral edges of the liquid film due to their higher effective evaporation area. The entire crystallization kinetics predicted by the simulation is plotted as the black curve in Figure 1G. It can be clearly seen that the simulation precisely captured the nucleation and steady growth stage, identifying the nucleation time at ∼300 s and a linear growth rate of 1.85 × 10^−4^–1.9 × 10^−4^/s (first derivatives of black simulation curve in Fig. 1G). This good match suggests that the continuous crystallization process can sustain a stabilized growth front despite the dynamic condition of the system [28, 29]. Therefore, if the steady solution feeding continues, the crystalline film would continuously grow at a constant rate leading to a roll‐to‐roll production.

### 2.2 Structure and Contents of Piezoelectric Film

**FIGURE 2:**
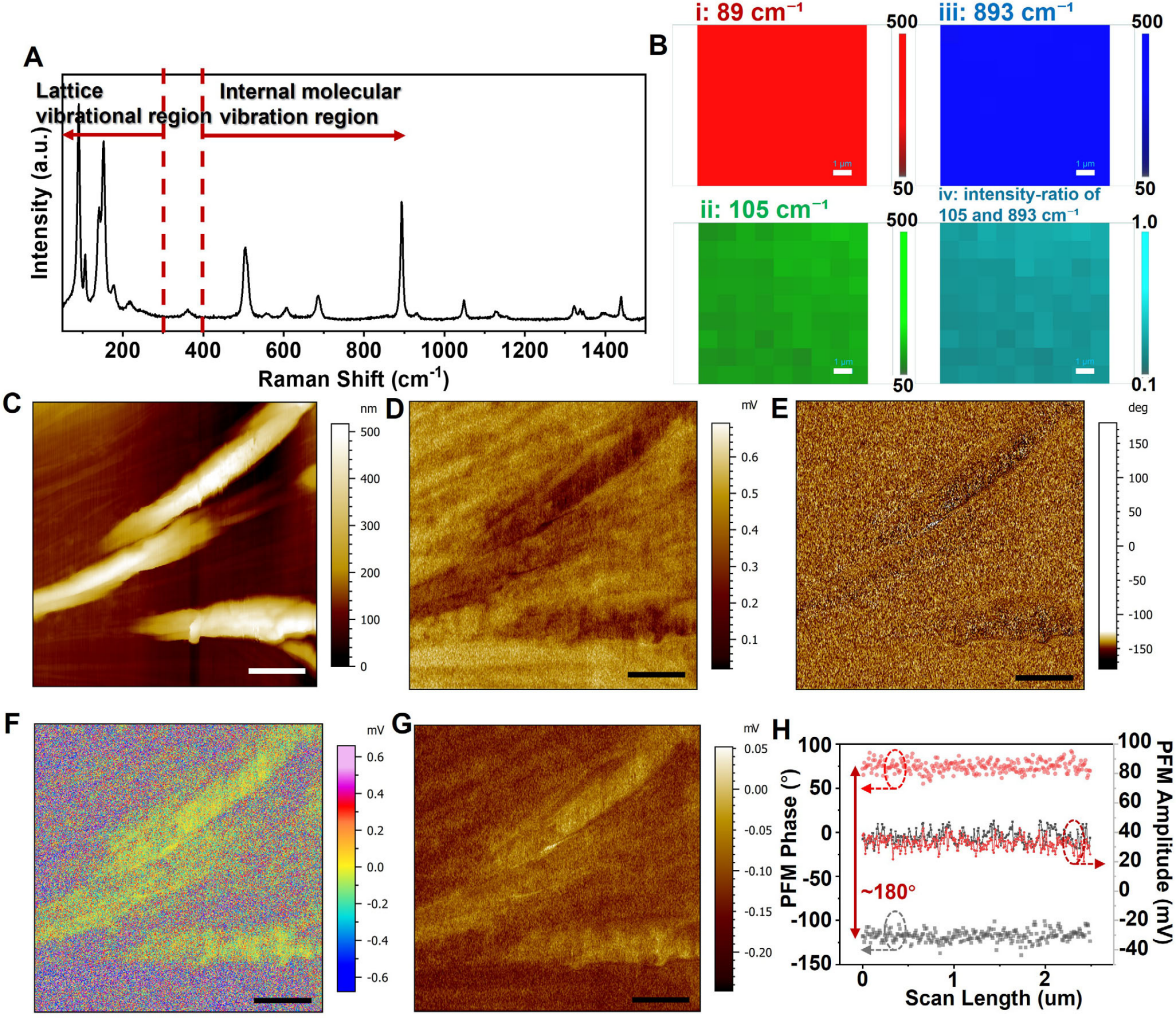
Characterization of the glycine‐PVA film. (A) Representative Raman spectrum showing the low‐frequency lattice vibrational region (<250 cm^−^^1^) and the internal molecular vibration region (>400 cm^−^^1^). The observed vibrational features are consistent with γ‐glycine. (B) Raman intensity maps of representative γ‐glycine modes, including a lattice vibration at approximately 89 cm^−^^1^ (i), a second low‐frequency lattice mode at approximately 105 cm^−^^1^ (ii), and an internal molecular vibration at approximately 893 cm^−^^1^ (iii), collected over a 10 × 10 µm^2^ area. (iv) Intensity‐ratio map between the 105 and 893 cm^−^^1^ modes (intensity at 105 cm^−^^1^: intensity of 893 cm^−^^1^), showing a uniform spatial distribution and confirming homogeneous γ‐glycine phase formation across the mapped region. Scale bars: 1 µm. (C) PinPoint‐PFM topography showing heterogeneous lamellar morphology. (D) PinPoint‐PFM amplitude and (E) phase images at 0 V DC exhibit spatially heterogeneous electromechanical contrast without clear domain structure. (F) PFM amplitude real part, while (G) PFM‐ amplitude imaginary part/ PFM quad. The scale bars of (C‐G) are 1 µm. (H) On‐field CR‐PFM line profiles at ±1 V DC show a consistent ∼180° phase offset with comparable amplitude levels. The red solid lines represent the PFM amplitude measured under a −1 V DC bias, while the red dots correspond to the PFM phase at −1 V DC. The black solid lines represent the PFM amplitude measured under a +1 V DC bias, and the black dots correspond to the PFM phase at +1 V DC.

Raman spectroscopy was used to determine the crystallographic phase of glycine formed on the PVA substrate and to assess its lateral uniformity. A representative Raman spectrum collected from a single location is shown in Figure 2A, while the lateral phase consistency was evaluated by Raman mapping over a 10 × 10 µm^2^ area using 81 measurement points (Figure 2B). In the low‐frequency lattice region (50–250 cm^−^^1^), the Raman spectrum exhibits prominent bands at approximately 89, 106, 141, and 151 cm^−^^1^, which closely match reported lattice modes of γ‐glycine [30]. These low‐frequency vibrations arise from collective lattice motions and are highly sensitive to polymorphic structure, providing clear evidence for γ‐glycine formation. In the mid‐ to high‐frequency region (400–1500 cm^−^^1^), characteristic γ‐glycine vibrational modes are observed at approximately 505, 689, 893, 1048, 1322, 1338, and 1439 cm^−^^1^, in agreement with literature assignments [30]. The γ‐glycine signatures appear with small, systematic peak shifts of 2–8 cm^−^^1^ relative to bulk reference values. These shifts occur without peak splitting or the emergence of additional bands, indicating that the underlying crystal structure remains unchanged. Instead, the shifts are attributed to local environmental effects, including polarization and hydrogen bonding at the interface with the hydroxyl‐rich PVA matrix, which can slightly perturb vibrational energies while preserving the γ‐glycine phase. The lateral uniformity of the γ‐glycine phase was confirmed by Raman mapping. Intensity maps of representative γ‐glycine modes, including a lattice vibration at approximately 89 cm^−^^1^, a second low‐frequency mode at approximately 105 cm^−^^1^, and an internal molecular vibration at approximately 893 cm^−^^1^, are shown in Figure 2Bi‐iii. All three maps display similar spatial distributions, with only minor intensity variations attributed to surface roughness, thickness variations, or small focus changes rather than chemical heterogeneity. An intensity‐ratio map between the 105 and 893 cm^−^^1^ modes (Figure 2B‐iv) is essentially featureless, further confirming homogeneous γ‐glycine phase formation across the mapped region.

The local electromechanical behavior of the glycine‐PVA composite was investigated using piezoresponse force microscopy (PFM). Because the polymer‐supported film is mechanically heterogeneous, conventional contact‐mode PFM provided limited and unstable contrast. PinPoint‐PFM was therefore employed to enable controlled indentation‐based measurements and improved signal reproducibility. PinPoint‐PFM topography acquired at 0 V DC is shown in Figure 2C, revealing a heterogeneous lamellar morphology consistent with γ‐glycine crystallites embedded within the PVA matrix. The corresponding amplitude image (Figure 2D) exhibits spatially heterogeneous contrast with signal levels in the several‐hundred‐microvolt range, indicating locally varying electromechanical coupling. From the topography images, a small number of γ‐glycine crystals are observed protruding from the surface. These features likely arise from localized concentration saturation during crystallization, leading to outgrowth from the PVA matrix. Notably, these protruding crystals exhibit reduced PFM amplitude contrast compared to the surrounding regions. This lower apparent response is likely influenced by differences in local contact mechanics, stiffness, and tip–sample coupling, rather than solely reflecting the intrinsic piezoelectric properties of γ‐glycine. In contrast, γ‐glycine domains embedded within the PVA matrix show relatively higher amplitude signals, which may be associated with improved mechanical coupling and more efficient electromechanical transduction. The phase image (Figure 2E) displays non‐binary contrast without well‐defined domain boundaries, with regions characterized by varying average phase offsets. Building on this, the reconstructed PFM real images (Figure 2F), calculated as amplitude × cos(phase), show a similar spatial trend. The corresponding imaginary (quadrature) component (Figure 2G), calculated as amplitude × sin(phase), further complements this representation. The protruding γ‐glycine crystals exhibit an enhanced quadrature response, which may indicate contributions from charge accumulation and/or viscoelastic phase lag, particularly in regions with reduced mechanical constraint from the surrounding matrix. Together, these reconstructed vector components highlight spatial variations in the electromechanical response across the composite surface, reflecting the combined influence of intrinsic material properties and the local mechanical environment.

Additional insight was obtained from contact‐resonance PFM (CR‐PFM) measurements performed on‐field at +1 V and −1 V DC bias with an AC excitation of 2 V. As shown in Figure 2H, the PFM amplitude remains comparable for both bias polarities, while phase line profiles exhibit values of approximately −120° and +70°, corresponding to an effective ∼180° phase inversion after phase wrapping. Although absolute phase values in CR‐PFM are influenced by resonance‐related phase rotation, the combination of symmetric amplitude and polarity‐dependent phase inversion provided independent confirmation of a sign change in the electromechanical response.

Taken together, Raman spectroscopy and PFM provide a consistent multiscale picture of γ‐glycine crystallized on a PVA substrate. Raman analysis confirms homogeneous γ‐glycine phase formation across the film, with small and spatially uniform peak shifts attributed to substrate‐induced polarization and interfacial interactions. These effects are consistent with the bias‐dependent electromechanical response observed by PFM, including reproducible phase inversion in CR‐PFM measurements. Collectively, the results indicate that the intrinsic piezoelectric response of γ‐glycine is locally expressed within the composite while being subtly modulated by interactions with the surrounding polymer matrix.

### 2.3 Evaluation of Large‐Scale Uniformity

**FIGURE 3:**
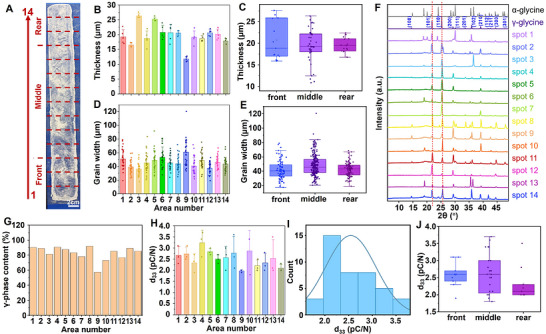
Evaluation of large‐scale uniformity. (A) Glycine–PVA film image showing the 14 areas on a 35‐cm‐long film used for structural and performance measurements. (B) The average thickness of 14 areas on the glycine‐PVA film. (C) Comparison of the thickness from front to rear regions in 14 areas of glycine‐PVA film. The box plot shows that medians (center line) of front, middle, and rear area are 18.86, 19.32, 19.48, mean values (open square) are 20.72(n = 15), 19.42(n = 40), 19.53(n = 15). (D) The average grain width of 14 areas on the glycine‐PVA film. (E) Comparison of the grain width from front to rear regions in 14 areas of glycine‐PVA film. The box plot shows that medians (center line) of front, middle, and rear area are 40.61, 45.62, 43.34, mean values (open square) are 42.13(n = 90), 48.39(n = 240), 42.40(n = 90). (F) XRD spectra of 14 areas of the glycine‐PVA film; Red dish lines mark the faces of (101) and (110) in γ‐phase glycine. (G) Fraction of γ‐phase glycine of 14 areas calculated from linear fitting of peak‐area ratio vs. fraction of γ‐glycine. (H) The average d_33_ of 14 areas on the glycine‐PVA film. (I) Statistical histogram of total‐area d33 for glycine‐PVA film. (J) Comparison of the d33 values from front to rear regions in 14 areas of glycine‐PVA film. The statistical descriptions of (B, D, H) were included in Table S2. The box plot shows that medians (center line) of front, middle, and rear area are 2.6, 2.6, 2.1, mean values(open square) are 2.58(n = 9), 2.62(n = 24), 2.32(n = 9). In the box plots (C,E,J), the box represents the interquartile range (IQR, 25th–75th percentiles), and the whiskers represent 1.5×IQR.

 To evaluate the large‐scale uniformity of our film's characteristics, a 35 cm × 5 cm film was divided into 14 different areas along the length direction (Figure 3A). Structural and piezoelectric characteristics were measured in every area from several random sampling points. The film thickness of each area was obtained from five randomly selected points on the SEM cross‐section images. As shown in Figure 3B, the thickness ranges from 10.9 to 27.6 µm and most areas clustered closely around an average value of 19.72 µm, indicating a reasonably good uniformity across the film. As shown in Figure 3C, the front, middle, and rear regions of the film exhibited comparable median values. The front region had a relatively higher average thickness and a broader distribution, while the middle region exhibited a wider overall spread. Both the middle and rear regions had a narrow distribution around the mean thickness (19.72 µm). As a characteristic to show the crystallization quality, glycine crystal grain width was measured by selecting 30 random points per area from the optical microscopy images (Figure S4). As shown in Figure 3D, the grain width ranges are from 17 to 120 µm and the mean values of most areas fall within the narrow range of 40–50 µm. Analysis of the grain width in the front, middle, and rear regions of the film (Figure 3E) revealed that all the three regions exhibited comparable median values. The middle region had a slightly higher grain‐width distribution with a few high‐value outliers. This behavior might be attributed to the fact that a fraction of grains in the middle region ceased growth as they intersected with each other, leading to a reduced grain density and a relatively enhanced lateral growth rate of the remaining grains [31, 32]

The crystalline phase distribution was identified by X‐ray diffraction (XRD) patterns taken at each region (Figure 3F) and compared with the reference spectra of γ‐glycine and α‐glycine are provided at the top. The broad peak at 19.7° corresponds to PVA, confirming its very low crystallinity. All measured areas exhibited the characteristic γ‐glycine diffraction peaks at 21.8° (101) and 25.3° (110). The XRD patterns from different areas also showed small variations in their dominant diffraction peaks. Due to heterogeneous nucleation and local variations in substrate morphology and liquid film curvature, the crystal orientation was not uniform across the film; however, the consistent presence of the out‐of‐plane [101] orientation ensured a robust piezoelectric response. The dominant out‐of‐plane orientation of the glycine films remained consistent across most regions, except the beginning and end regions, suggesting most of the film had closely aligned crystallography orientation. However, the dominant out‐of‐plane orientation of the glycine films remains consistent across most regions, except the beginning and end regions, suggesting that most of the film had closely aligned crystallography orientation. Small peaks from α‐glycine could also be observed from most regions except spots 4 and 5. To further quantify the phase composition, Raman spectroscopy was employed to evaluate the relative γ‐glycine content in each area. Compared to XRD, Ramen spectra rely on characteristic vibrational modes of different phases to quantify the ratio of γ phase over other non‐piezoelectric phases, providing a more practical and consistent measurement for our system. As shown in Figure S5, the γ‐glycine exhibits a characteristic absorption band in the 1330–1360 cm^−^^1^ region, while the α‐glycine shows a distinct band at 1445–1470 cm^−^^1^. Based on these two polymorph‐specific peak areas, the fraction of γ‐form glycine in a mixed α/γ sample was quantified based on the peak area ratio (Figure S8), and the calculated γ‐phase fractions in the 14 areas are shown in Figure 3G. Most areas exhibit a γ‐phase fraction of approximately 80%. The only reduced γ‐glycine fraction identified at region #9 was due to process perturbation from a reduced solution supply, leading to a reduced kinetic window for γ‐glycine formation [33, 34]. Compared to static growth, the dynamic conveyor process involved rapid crystallization, evaporation‐induced concentration fluctuations, and substrate motion, which collectively limited crystal growth and alignment, reduced crystallinity, and led to smaller domains, thereby resulting in slightly lower piezoelectric performance.

Eventually, the bulk piezoelectric property of the film was characterized by measuring the d_33_ coefficients, which were collected and averaged from three different spots within each area (Figure 3H, Figure S9, Table S1). The values across the entire film fell within 1.8–3.7 pC/N, and most areas exhibited values centered around 2.5 pC/N. These values were lower than the maximum outcome achieved from static growth conditions. This discrepancy could be attributed to the variations in grain size and thickness uniformity, and a slightly lower γ‐phase content as revealed in morphological and phase analyses. Statistical analysis of all the d_33_ values measured from all areas (Figure 3I) showed an average d_33_ value of 2.545 pC/N, with most measurements centering around this value. The regional variations of d_33_ were analyzed using box plots corresponding to different spatial positions on the film (Figure S10 and Figure 3J). While the average values were close, the central region of the film displayed a much more uniform piezoelectric property compared to the edge regions (∼1 cm from each side). In addition, the front and rear regions exhibit narrower distributions than the middle region, which could be attributed to the broader distribution of the fraction of γ‐glycine in the middle region (from spots #4‐#11). The median values in the front and middle regions are close to the mean values from the statistical histogram. The outlier with the lowest average d_33_ value of 1.97 pC/N (spot 9) was due to the film quality induced by process perturbations. The other deviations on thickness, grain width and composites observed at spot #9 may result from local structural heterogeneity, minor surface defects caused by recrystallization, or small undulations of the PET substrate.

As discussed above, local fluctuations during the continuous conveyor process, especially at the film edges, may induce non‐uniform crystal alignment, leading to variations in crystallographic orientation and defect formation, and thus causing variations in piezoelectric properties. Nevertheless, the performance remains largely uniform across the film. Overall, the as‐received glycine‐PVA biocrystal film displays acceptable uniformity and homogeneous in terms of geometry, phase, and piezoelectric responses. This process has been performed eighteen times and the obtained films exhibited consistent crystal growth behavior as well as reproducible properties (Figures S11 and S12), demonstrating its potential for scalable production.

### 2.4 Mass Fabrication and Operation of Piezoelectric Devices

**FIGURE 4:**
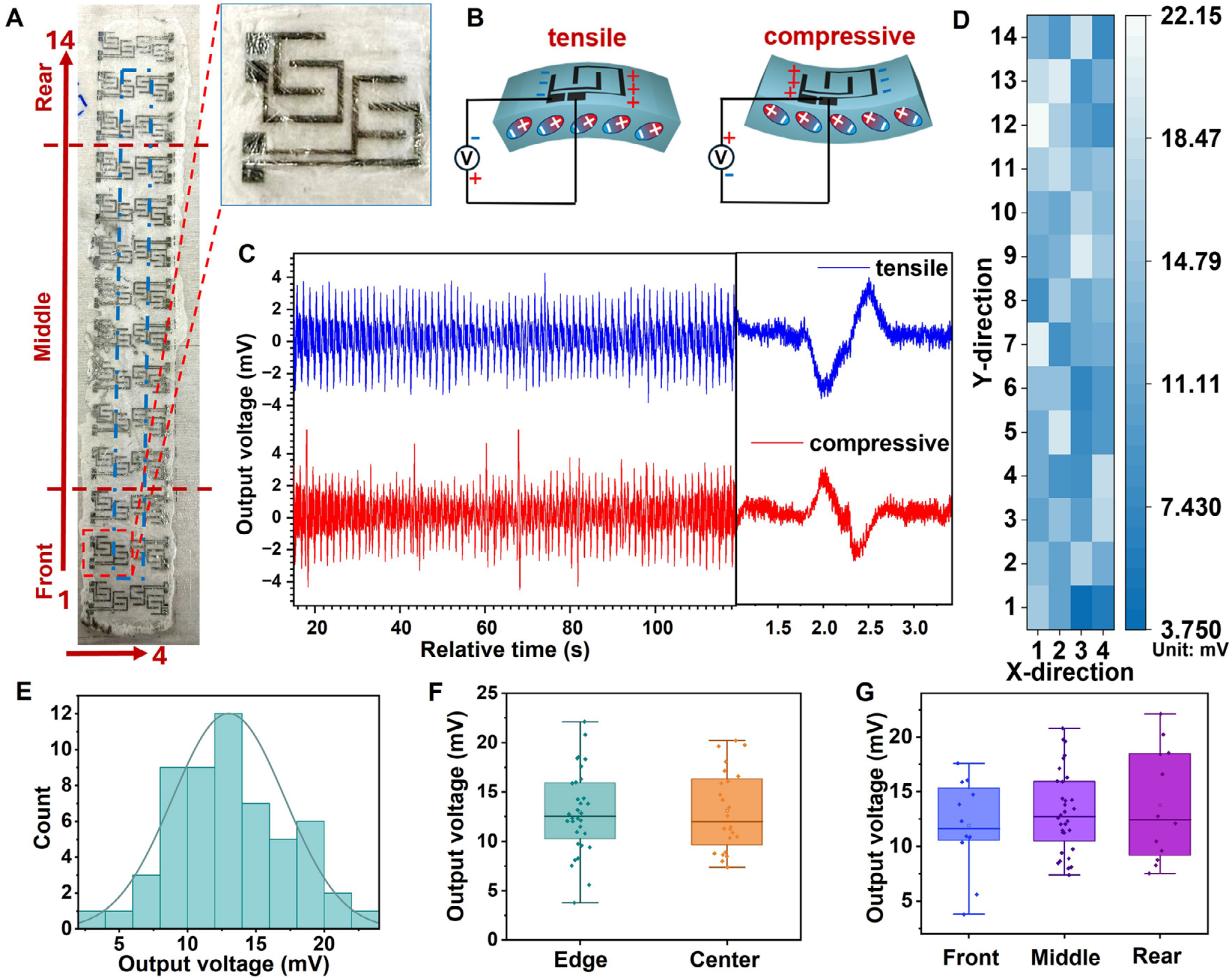
Mass production of piezoelectric devices. (A) Photograph of a 30‐cm‐long and 5‐cm –wide glycine‐PVA film with 56 9.5 mm × 8.5 mm printed piezoelectric surface devices. Inset shows the surface electrodes arrangement for two devices. (B) Schematics of the dipole rotation process and charge distribution of glycine‐PVA films under tensile or compressive stresses. (C) Piezoelectric voltage output of glycine‐PVA films under tensile or compressive stresses at 0.67 Hz. Right insets are the enlarged one peak showing the opposite polarization under different strain conditions. (D) The spatial mapping of average piezoelectric voltage output within 30 s of 56 independent devices. (E) Statistical histogram of output voltages for 56 devices on glycine‐PVA film. (F,G) Comparison of the output voltage values from center to edge and front to rear devices on the piezoelectric biocrystal film. Box plots show that medians (center line) of edge, center, front, middle, rear area are 12.53, 12.02, 11.62, 12.72, 12.43, mean values (open square) are 12.97(n = 32), 13.08(n = 24), 11.90(n = 12), 13.15(n = 32), 13.78(n = 12). In the box plots (F,G), the box represents the interquartile range (IQR, 25th–75th percentiles), and the whiskers represent 1.5×IQR.

To demonstrate the practical value of the piezoelectric biocrystal film from this scalable process, multiple piezoelectric nanogenerators (NGs) were fabricated on a 30 cm × 5 cm biocrystal film using inkjet printing. Each device compromised a pair of interdigitated surface electrodes with an electrode spacing of 7.5 mm, covering a total surface area of 80.75 mm^2^ (inset of Figure 4A). With this configuration, we were able to print four devices along the width direction and 14 rows of devices along the length of the film with all their electrode leads aligned on both sides for electricity output (Figure 4A). This electrode design was intended to demonstrate the feasibility of mass device fabrication together with local piezoelectric response characterization from surface strains. The devices were tested as a group using a custom‐designed four‐point bending apparatus with replaceable modules featuring asymmetric upper and lower span lengths (Figure S13). This setup introduced homogeneous strain to the electrode side of the film, given the large thickness difference between the glycine film and the substrate (0.62 mm). As such, tensile or compressive strain could be uniformly applied to the glycine film when the device was bent upward or downward, respectively (Figure 4B). The corresponding in‐plane piezoelectricity outputs under such conditions were monitored from the two electrodes. Figure 4C shows the typical voltage output from a single device when it was continuously bent over 70 cycles. The output amplitudes remained at a consistent level with a peak‐to‐peak voltage (V_pp_) of ∼6 mV (top curve). While the device was bent toward opposite direction, similar series of voltage output was generated with a comparable V_pp_ amplitude. Enlarged voltage profile (right panel of Figure 4C) shown that as the film was bent upward, negative voltage signal was produced first followed by a positive peak as the film is relaxed. This corresponded to the tensile strain subjected by the piezoelectric biocrystal. As the film was bent downward, compressive strain induced positive voltage first showing a mirrored profile from the upward bending. This output characteristic confirms that direct correlation between the voltage output and the film's in‐plane piezoelectric responses. For comparison, a control experiment was performed on a pure PVA film‐coated PET substrate, followed by the same electrode fabrication and electrical measurement under identical conditions. The resulting voltage was mostly noise‐level signal without exhibiting the characteristic periodic response or distinct peak features (Figure S15). These results further confirmed that the device output was originates from the piezoelectric response of the glycine–PVA film.

To further assess the device‐to‐device uniformity, all 56 devices were measured under identical deformation conditions. The average V_pp_ over 30 s was calculated for each device and displayed in a spatial heat map (Figure 4D). All the devices exhibited steady and detectable piezoelectric output, whereas the average V_pp_ varied in a relatively large range from 3.75 to 22.15 mV. The stable output voltage of every independent device highlighted the robustness of our large‐area fabrication method. Analysis of the spatial distribution of the output voltage was performed by constructing a statistical histogram over all 56 devices (Figure 4E), which yielded output voltage in the region of 3.79–22.12 mV. Most measurements are concentrated around the mean value of 13.02 mV with a moderate spread. This relatively low output voltage was partially due to the surface electrode configuration, which only collected charges from approximately one‐third of the active area without poling. As shown in the box plots in Figure 4F, the trend of output voltage was similar to that observed for the piezoelectric coefficient. The devices on both edge areas exhibited a broader distribution of V_pp_ than the devices in the central region, while the median values in both cases remained close to the corresponding mean value in histogram. A comparison of devices along the length direction (Figure 4G) further revealed that the rear region had a wider box distribution, consistent with more scattered d_33_ values observed in the same region. Devices located in the middle region consistently exhibited the most stable and uniform piezoelectric outputs with an average value of 13.15 mV. Despite the two devices at front region that showed the lowest output at 3.79 and 5.6 mV (possibility due to the local defects formed at the beginning of the fabrication process), the rest of output from 54 devices all generated outputs more than 7 mV. The output distribution was consistent with the observed spatial uniformity of piezoelectric performance over a broad central region of the film, validating the potential of a scalable strategy for mass production of piezoelectric devices.

## 3 Conclusions

In summary, we demonstrated a scalable and cost‐effective manufacturing strategy for continuously producing glycine‐based piezoelectric biocrystal films. The continuous crystallization of glycine‐PVA film was achieved by dynamic balancing the feeding rate and evaporation speed of the precursor solution. This well‐controlled crystallization kinetics was validated by well‐matched experimental observations with continuum‐scale kinetic modeling. Raman mapping analysis confirmed homogeneous γ‐glycine phase formation across the film, which was consistent with the bias‐dependent electromechanical response observed by PFM, including reproducible phase inversion in both SS‐PFM and CR‐PFM measurements. The as‐fabricated piezoelectric films exhibited excellent macroscale uniformity with thickness of ∼20 µm, grain width of ∼45 µm, and d_33_ of ∼2.5 pC/N. The film exhibits a predominantly high γ‐phase content with most measured regions exceeding 80%, providing a structural basis for the relatively uniform distribution of functional characteristics and piezoelectric response, thereby supporting the robustness of the process for producing functional piezoelectric films at scale. 56 piezoelectric surface devices were fabricated on a biocrystal film. All of them showed stable output voltages under a series of bending actions, confirming the reliability and potential for mass device production of our continuous fabrication approach. To further improve the uniformity in large‐scale production, several key factors should be considered. Particularly, precise coordination of solution feeding and substrate transport is critical to stabilize the crystallization front during the continuous process. Meanwhile, simultaneous control over evaporation and diffusion, along with stable thermal condition, is also essential for maintaining consistent growth and improving the overall orientation uniformity of the biocrystal film. With their intrinsic biocompatibility, mechanical flexibility, scalable manufacturability, and compatibility with roll‐to‐roll systems, such piezoelectric biocrystal films hold strong potential for applications in wearable electronics, self‐powered sensing systems, and bio‐integrated devices. Overall, this study established a dynamically controlled crystallization framework enabling continuous and scalable biocrystal film growth by coupling solution transport, thermal gradients, and substrate movement. It offered a pathway leading from conventional lab‐based static crystallization processes to industrially applicable manufacturing applications. Incorporating real‐time feedback mechanisms to couple the crystallization rate and local conditions would eventually automate the process, enabling a large‐scale continuous biocrystal manufacturing process.

## 4 Experimental Section

### 4.1 Fabrication of Glycine–PVA Films

PVA water solution (10% w/v) was prepared by dissolving 10 g PVA (Mw 13 000–23 000) in 100 mL deionized water. Glycine solution (10% w/v) was prepared by dissolving 10 g glycine in 100 mL deionized water. 30 mL of 10% glycine solution was added into 10 mL of 10% PVA solution to obtain a glycine–PVA solution with glycine‐to‐PVA ratio of 3 : 1. A homogeneous solution was obtained by stirring the mixture for 1 h. The as‐prepared solution was then directly used for film growth. The scalable manufacturing system is designed based on a flat belt conveyor apparatus driven by a DC gear motor (Dayton, model 1LPW5), controlled by a DC speed controller (Dayton, model 4Z527H), which allowed continuous adjustment of the motor speed. A 5‐mil polyester (PET) film as a substrate, which was held and transported by a conveyor. The mixed solution of glycine and PVA was added by a combination of a burette and a PLA solution dispenser with a 4 cm‐width narrow line‐shaped outlet. In simulation, the rate of solution feeding was set to 0.2 mL/min, and the speed of the conveyor was set to 0.35 cm/min. In the fabrication process, the rate of solution feeding and speed of conveyor can be adjusted within the region of 0.15–0.3 mL/min and 0.35–1.2 cm/min, respectively, according to the target thickness of glycine‐PVA films. A 1050 W infrared heater was placed 27 cm in front of the dispenser. The blade system has effective width of 10 cm with adjustable height from 0–3500 µm. The solution entering the heated region would evaporate and glycine crystal grew along the direction of substrate transportation. Finally, a large‐scale piezoelectric film with continuous crystallized region was fabricated. The as‐received glycine–PVA films could be directly peeled off from the substrates at room temperature in atmosphere for characterizations of Raman spectra, PFM, SEM, d_33_, and XRD.

### 4.2 Structure and Morphology Characterization

SEM observations of the cross‐section of glycine–PVA composite film were performed on a Zeiss GeminiSEM 450 field‐emission microscope. X‐Ray diffraction pattern of glycine–PVA composite films was acquired from the Bruker D8 Discovery with Cu K‐alpha radiation. Polarized optical images were performed on a Nikon Optiphot microscope equipped with AmScope MW1000 10 MP color CMOS camera. Raman spectra were detected by LabRAM HR Evolution Confocal Raman Microscope using 532 nm laser source. The standard samples with a series fraction of γ‐form glycine were made by mixing α‐form and γ‐form glycine in different ratios. The Raman mapping measurements were acquired using a LabRAM HR Evolution system (HORIBA, Japan) under a 785 nm laser source, and spectral processing and mapping analysis were performed using LabSpec 6 software. The thickness and grain width values were measured by Image J. Local electromechanical behavior was examined using piezoresponse force microscopy (PFM) performed on an NX‐10 atomic force microscope (Park Systems, South Korea). A conductive PPP‐EFM probe (Nanosensors) was used throughout the study, with a nominal spring constant of 2.8 N/m. The cantilever spring constant was calibrated prior to measurement using the thermal tune method.

PFM was conducted in off‐resonance vertical PFM mode using an excitation frequency of 17 kHz, while switching spectroscopy PFM (SS‐PFM) measurements were performed under contact‐resonance (CR) conditions to enhance signal sensitivity. The CR‐PFM performance was performed by tracing the differences between the CR frequency to the cantilever free resonance frequency. For measurements on the mechanically heterogeneous glycine‐PVA composite surface, PinPoint‐PFM was employed. In this mode, the PFM signal is acquired during controlled tip–sample indentation at each pixel using an AC drive voltage of 2 V and an indentation depth of approximately 30–50 nm, improving contact stability and reducing lateral‐force‐related artifacts. Bias‐dependent measurements were carried out by applying a DC voltage sweep to the conductive AFM tip while recording the PFM amplitude and phase response. Additional box‐writing experiments were performed using off‐resonance PFM to probe localized electromechanical behavior under applied bias. Each specimen was mounted on an AFM sample puck using conductive carbon tape, and a small amount of silver paste was applied along the sample edge to ensure stable electrical grounding during scanning probe measurements. All AFM and PFM images were pre‐processed using MountainSPIP software, including background flattening and line‐by‐line artifact removal.

### 4.3 Fraction Measurement of γ‐Glycine

The peak area ratio of two polymorph‐specific peak areas was calculated by the equation:

(1) $$Arearatio=\frac{Area_{1330-1360_{cm^{-1}}}}{Area_{1330-1360_{cm^{-1}}}+Area_{1445-1470_{cm^{-1}}}}$$

This equation was adopted from previous study [35], with the integration range slightly adjusted to account for the peak position shifts observed in our experimental Raman spectra. The fraction of γ‐form glycine in mixed α/γ samples was quantified using a linear calibration between the Raman peak area ratio and the polymorphic fraction. The calibration curve is presented in Figure S7, and a linear fit with R^2^ = 0.9867 was obtained. This calibration was subsequently applied to estimate the γ‐form fraction across different areas of the film. The α‐form and γ‐form glycine crystals were obtained following a previously established protocol [36].

### 4.4 Kinetics Process Measurement

The optical images and the video of the film growth process were taken with a phone. Then pictures of films were exported at every 2.5 min. The transition fractions (*X*) were calculated from the measured film sizes by Image J:X =  *A*_c_/*A*_0_ where *A*_c_ is the size of the crystallized area, and *A*_0_ is the size of the whole film. Our previous work revealed that the intact crystalline zones had no residual solution in the crystals, and the transition zone between crystalline and liquid regions extending only ∼10 µm at the growth front. Therefore, the uncertainty in the area measurement can be ignored.

### 4.5 Piezoelectric Device Fabrication and Characterization

For measurements of voltage and current output, small electrodes (9.5 mm × 8.5 mm) were printed on the whole films by inkjet Dimatix Materials (DMP‐2850) printer with a 2.4‐pL (picoliter) printhead and silver nanoparticle ink (Ag NP) procured from Novacentrix Inc. Then the devices were connected through conductive wire for testing. The force was applied to the devices by a computer‐controlled actuator (LinMot E1100) at 0.67 Hz with an amplitude of 1.2 cm. A smaller module was fixed onto a program‐controlled actuator that achieved repeatable film bending through precisely controlled vertical extrusion, while a bigger module was at the bottom. The piezoelectric voltage outputs were recorded by connecting probes of a multimeter (DMM 6500, Keithley, internal resistance 10 Mohm) to the device. The piezoelectric *d*_33_ coefficients were measured by a quasi‐static piezoelectric constant *d*_33_ meter (PKD3‐2000‐F10N, PolyK Technologies, LLC) under forces in the range of 3–5 N.

## Conflicts of Interest

The authors declare no conflict of interest.

## Supporting information




**Supporting File 1**: smll74257‐sup‐0001‐SuppMat.docx.

**Supporting File 2**: smll74257‐sup‐0002‐MovieS1.mp4.

## Data Availability

The data that support the findings of this study are available from the corresponding author upon reasonable request.
