# Supplementary material for: Scalable Manufacturing of Amino Acid‐Based Piezoelectric Biocrystal Films
Source: Small. 2026 Jun 21;22(45):e74257. doi: 10.1002/smll.74257 (PMC13472288; doi:10.1002/smll.74257)
Supplement: Supplementary file 1 [file SMLL-22-e74257-s001.docx]

**Supporting Information**

**Scalable Manufacturing of Amino Acid-Based Piezoelectric Biocrystal Films**

Shuting Wang,^1^ Bijay Dhungana,^2^ Anirudh Reddy Bendaram,^3^ Jiajie Sui,^1^ Chenli Jia,^1^ Fnu Joshua^4^, Craig Neal,^2^ Ruoxing Wang,^1^ Pengfei Chen,^1^ Fengdan Pan,^1^ Sharar Muhtasim^7^, Joseph Andrews^3,7^, Wenxiao Pan,^3^ Lei Zhai^4,5^, Sudipta Seal,^2,5,6^ Lih-Sheng Turng,^3,*^ Xudong Wang^1,*^

1. Department of Materials Science and Engineering, University of Wisconsin-Madison, Madison, WI, 53706, USA
2. Advanced Materials Processing and Analysis Center (AMPAC), Department of Materials Science & Engineering, University of Central Florida, Orlando, FL 32816, USA
3. Department of Mechanical Engineering, University of Wisconsin-Madison, Madison, WI 53706, USA
4. Department of Chemistry, University of Central Florida, 4000 Central Florida Blvd., Orlando, FL 32816, USA
5. Nanoscience and Technology Center, University of Central Florida, 12424 Research Parkway, Orlando, FL 32826, USA
6. Biionix cluster, College of Medicine, University of Central Florida, Orlando, FL 32816, USA
7. Department of Electrical and Computer Engineering, University of Wisconsin-Madison, Madison, WI, 53706, USA

**This file includes:**

Supplementary Text

Figs. S1 to S15

Table S1 to S3

Caption for Movie S1

References

**Supplementary Text**

Coupled evaporation-diffusion process in simulation

To clarify the coupled evaporation–diffusion model, some critical state variables are utilized to describe the governing equation. $u_{i}(x,t)$ is the solute concentration of glycine and PVA; $\theta_{L}(x,t)$ is the local liquid volume fraction; $T\left( x,t \right)$ is the temperature field.

Solute transport is described by the diffusion–reaction equation:

$$\begin{aligned} \frac{\partial u_{i}}{\partial t}=D_{i}\left( T \right)\nabla^{2}u_{i}-\frac{\beta}{\theta_{L}}\frac{\partial\theta_{L}}{\partial t}u_{i},\#\left( 1 \right) \end{aligned}$$

with zero-flux boundary conditions at the solid walls. Here $D_{i}\left( T \right)$ are the temperature-dependent diffusion coefficients of glycine and PVA which can be extrapolated from Stokes–Einstein relation, β is a coupling constant calibrated against laboratory-scale crystallization data. $\theta_{L}$ obeys a diffusion-type equation:

$$\begin{aligned} \frac{\partial\theta_{L}}{\partial t}=D_{L}\left( \theta_{L} \right)\nabla^{2}\theta_{L}, in \Omega,\#\left( 2 \right) \end{aligned}$$

with boundary condition:

$$\begin{aligned} \boldsymbol{n}\cdot\left( D_{L}\nabla\theta_{L} \right)=\frac{J}{\rho_{L}}, on \partial\Omega,\#\left( 3 \right) \end{aligned}$$

where $\rho_{L}$ is the liquid density. The liquid-phase diffusivity $D_{L}(\theta_{L})$ is defined as a simple saturation-dependent form^1^:

$$\begin{aligned} D_{L}\left( \theta_{L} \right)=\left\{ \begin{matrix} \alpha\left( \theta_{L}-{\theta^{*}}_{L} \right), & \theta_{L}\geq{\theta^{*}}_{L}, \\ 0, & \theta_{L}<{\theta^{*}}_{L}, \end{matrix} \right.\#\left( 4 \right) \end{aligned}$$

where ${\theta^{*}}_{L}$is a residual liquid fraction (below which liquid is trapped in the microstructure) and α is a proportionality constant.

The evaporative mass flux *J* at the air–liquid interface couples the gas phase to the liquid film:

$$\begin{aligned} J=-h_{m}\left( \rho_{sat}-\rho_{\infty} \right),\#\left( 5 \right) \end{aligned}$$

where $h_{m}$ is an effective mass-transfer coefficient computed from Sherwood-number correlations for flow over a flat plate, $\rho_{\infty}$ is the ambient vapor density, and $\rho_{sat}$ is the saturation vapor density above the solution*. J* is applied only on parts of the free surface where liquid is present (i.e. where $\theta_{L}$ > ${\theta^{*}}_{L}$).

The temperature field in the liquid region $\Omega_{L}\left( t \right)$ is obtained from a transient conduction equation:

$$\begin{aligned} \rho_{\mathrm{eff}}C_{p,\mathrm{eff}}\frac{\partial T}{\partial t}=\nabla\cdot\left( \lambda_{\mathrm{eff}}\nabla T \right), in \Omega_{L}\left( t \right),\#\left( 6 \right) \end{aligned}$$

closed with a Robin-type boundary condition at the free surface:

$$\begin{aligned} \boldsymbol{n}\cdot\left( \lambda_{\mathrm{eff}}\nabla T \right)=J\mathcal{L-}h\left( T-T_{room} \right),\#\left( 7 \right) \end{aligned}$$

where L is the latent heat of evaporation and *h* is an effective convective heat transfer coefficient.

In summary, evaporation, diffusion, and heat transfer are coupled through (i) the dependence of $D_{i}\left( T \right)$ and $D_{L}(\theta_{L})$ on *T* and $\theta_{L}$, (ii) the dependence of the evaporative flux *J* on *T*, and (iii) the pseudo-reaction term in **Eq. (1)**, which links solvent loss (through $\partial_{t}\theta_{L}$) to the evolution of solute concentrations to simulate crystallization process.

CR-PFM characterization of γ-glycine on PVA.

Bias-dependent behavior was examined using switching spectroscopy PFM (SS-PFM) under contact-resonance conditions with a DC voltage sweep from −5 to +5 V. A 9 × 9 SS-PFM array shows reproducible hysteretic amplitude and phase responses across the measured region.^2^ A representative amplitude and phase loop (Fig. S3A and S3B) exhibits a phase separation of approximately 170°, indicating a polarity-dependent electromechanical response corresponding to a sign change in the effective coupling under opposite electric field directions. Because these measurements were acquired on-field during DC bias application, electrostatic and interfacial contributions cannot be fully excluded. The SS-PFM results are therefore interpreted as evidence of bias-dependent electromechanical behavior rather than definitive nanoscale polarization switching.

This behavior does not indicate ferroelectric domain structure; rather, it reflects variations in local contact mechanics, stiffness, and electromechanical coupling across the composite surface. Accordingly, static PinPoint-PFM imaging is interpreted as qualitative evidence of spatially distributed electromechanical activity rather than direct proof of polarization orientation or domain switching.

**Supplementary Figs:**

Figure S1: Digital photograph of the setup of burette, solution dispenser and infrared heater surrounding the conveyor, showing the growing process of the glycine-PVA film on PET substrates.

Figure S2: Digital photograph of the fabrication setup with additional blade system.

Figure S3: CR-PFM characterization of γ-glycine on PVA. (A) CR-PFM amplitude shows a butterfly-like bias dependence, while (B) the corresponding phase loop displays an ~170–180° phase inversion.

Figure S4: Top-view optical microscopic images of area 1-14 (A-N) of the glycine-PVA film, showing the grain structure of glycine crystals.

Figure S5: Comparison of Raman shift characteristic relative peak intensities for the polymorphic mixture of α-glycine and γ-glycine at various γ-glycine weight fractions;

Figure S6: XRD of pure γ-glycine and α-glycine used to make standard samples with different fracture of γ-phase glycine. The standard XRD spectra of γ-glycine and α-glycine are given on top.

Figure S7: Standard calibration of γ-glycine characteristic peak area ratio from Raman shift and γ-glycine weight fraction in the polymorphic mixture of α-glycine and γ-glycine.

Figure S8: Raman shift of 14 areas on the glycine-PVA film, intensities of whose characteristic peaks were used to calculate Area ratios.

Figure S9: Digital images of the PVA-glycine film with labelled 42 points where d_33_ were measured in different 14 areas. Red labels show “center” and white labels show "edge”.

Table S1. d_33_ values measured from the PVA-glycine film in 14 areas with 3 points each. Unit: pC/N.

|  | Point 1 | Point 2 | Point 3 | Average |
| --- | --- | --- | --- | --- |
| Area 1 | 3.1 | 2.6 | 2.3 | 2.67±0.40 |
| Area 2 | 3.1 | 2.6 | 2.5 | 2.73±0.32 |
| Area 3 | 1.9 | 2.4 | 2.7 | 2.33±0.40 |
| Area 4 | 3.7 | 2.6 | 3.4 | 3.23±0.57 |
| Area 5 | 3.0 | 2.8 | 2.7 | 2.83±0.15 |
| Area 6 | 2.7 | 2.5 | 2.3 | 2.50±0.20 |
| Area 7 | 2.7 | 3.0 | 2.0 | 2.57±0.51 |
| Area 8 | 3.6 | 2.1 | 2.6 | 2.77±0.76 |
| Area 9 | 1.9 | 2.0 | 2.0 | 1.97±0.06 |
| Area 10 | 1.8 | 3.4 | 3.4 | 2.87±0.92 |
| Area 11 | 2.0 | 2.5 | 2.1 | 2.20±0.26 |
| Area 12 | 2.0 | 2.2 | 2.8 | 2.33±0.42 |
| Area 13 | 3.5 | 2.0 | 2.1 | 2.53±0.84 |
| Area 14 | 2.0 | 2.0 | 2.3 | 2.10±0.17 |

Figure S10: Comparison of the d33 values from center to edge regions in 14 areas of glycine-PVA film; The spots labeled in red in Fig. S9 are classified as the center region while in white are classified as the edge region. The box plot shows that medians (center line) of center, edge areas are 2.5, 2.65, mean values(open square) are 2.51(n=20), 2.58(n=22). In the box plot, the interquartile range (box) is 25–75 percentile, and whiskers is 1.5×IQR.

Figure S11: Typical images of Glycine–PVA films synthesized through additional processes showing good reproducibility of the process.

Figure S12: Characteristics of another glycine-PVA film: (A) Glycine–PVA film image showing the 11 areas on a 27-cm-long film used for structural and performance measurements. (B) The average grain width of 11 areas on the glycine-PVA film. (C) The average thickness of 11 areas on the glycine-PVA film. (D) The average d_33_ of 11 areas on the glycine-PVA film. (E) XRD spectra of 11 areas of the glycine-PVA film; Red dish lines mark the faces of (101) and (110) in γ-phase glycine. The statistical descriptions of (B-D) were included in **Table S3**.

Figure S13: Schematics of extrusion of custom-designed four-point bending setup equipped with top and bottom modules of different lengths. The top module was fixed onto a program-controlled actuator that achieved repeatable film bending with extrusion distance of 1.2 cm and frequency of 0.67 Hz.

Figure S14: Piezoelectric voltage output of representative devices from 14 electrode pair groups under compressive stresses at 0.67 Hz with the extrusion distance of 1.2 cm. The average magnitude of peak-to-peak voltage of each device is showed in Figure 4D.

Figure S15: Voltage output of PVA film under bending stresses at 0.67 Hz.

Table S2. Statistics description of thickness, grain width, d_33_ values measured from the PVA-glycine film in 14 areas in Figure 3(mean ± SD, n=5, 30, 3 for thickness, grain width, d_33_ values).

|  | Thickness (μm**)** | Grain width (μm**)** | d_33_ (pC/N) |
| --- | --- | --- | --- |
| Area 1 | 19.28±2.35 | 50.85±14.00 | 2.67±0.40 |
| Area 2 | 16.53±0.72 | 39.33±9.41 | 2.73±0.32 |
| Area 3 | 26.35±0.78 | 36.20±8.52 | 2.33±0.40 |
| Area 4 | 18.74±2.51 | 45.09±13.62 | 3.23±0.57 |
| Area 5 | 25.21±0.69 | 48.87±14.04 | 2.83±0.15 |
| Area 6 | 20.76±2.37 | 53.42±15.06 | 2.50±0.20 |
| Area 7 | 20.66±2.79 | 45.23±10.15 | 2.57±0.51 |
| Area 8 | 20.48±0.86 | 43.97±11.09 | 2.77±0.76 |
| Area 9 | 11.79±0.83 | 60.99±18.88 | 1.97±0.06 |
| Area 10 | 19.09±2.18 | 40.65±12.47 | 2.87±0.92 |
| Area 11 | 18.64±1.04 | 48.91±11.04 | 2.20±0.26 |
| Area 12 | 20.62±1.08 | 37.51±8.19 | 2.33±0.42 |
| Area 13 | 20.14±1.77 | 46.05±9.12 | 2.53±0.84 |
| Area 14 | 17.81±0.98 | 43.64±9.50 | 2.10±0.17 |

Table S3. Statistics description of thickness, grain width, d_33_ values measured from the PVA-glycine film in 11 areas in Figure S12(mean ± SD, n=5, 30 for thickness, grain width).

|  | Thickness (μm**)** | Grain width (μm**)** | d_33_ (pC/N) |
| --- | --- | --- | --- |
| Area 1 | 13.72±0.80 | 38.98±14.73 | 2.50±0.53 (n=7) |
| Area 2 | 14.06±0.81 | 70.42±25.17 | 3.20±0.98 (n=5) |
| Area 3 | 18.63±7.75 | 73.26±21.57 | 3.48±0.95 (n=5) |
| Area 4 | 29.88±5.59 | 67.06±26.97 | 3.98±1.14 (n=5) |
| Area 5 | 28.11±1.39 | 86.48±38.27 | 3.76±0.76 (n=5) |
| Area 6 | 27.95±1.19 | 114.18±63.53 | 3.15±1.03 (n=6) |
| Area 7 | 12.57±0.44 | 131.64±64.38 | 2.32±0.19 (n=5) |
| Area 8 | 24.59±1.33 | 97.72±61.33 | 2.70±0.80 (n=6) |
| Area 9 | 17.51±0.92 | 113.23±68.56 | 2.56±0.78 (n=5) |
| Area 10 | 30.78±4.77 | 82.87±35.56 | 3.50±1.24 (n=6) |
| Area 11 | 29.94±2.78 | 72.95±30.61 | 3.48±1.45 (n=9) |

**Supplementary Movie:**

**Movie S1:** The fabrication process of a 30 cm–long glycine–PVA film on a PET substrate. The total growth duration was 63 min. To visualize the continuous growth behavior, the video was assembled from 10 s segments recorded every 3 min during the fabrication process.

**References:**

(1)	Murru, M.; Giorgio, G.; Montomoli, S.; Ricard, F.; Stepanek, F. Model-Based Scale-up of Vacuum Contact Drying of Pharmaceutical Compounds. *Chem. Eng. Sci.* **2011**, *66* (21), 5045–5054. https://doi.org/10.1016/j.ces.2011.06.059.

(2)	Jesse, S.; Baddorf, A. P.; Kalinin, S. V. Switching Spectroscopy Piezoresponse Force Microscopy of Ferroelectric Materials. *Appl. Phys. Lett.* **2006**, *88* (6), 062908. https://doi.org/10.1063/1.2172216.
